# Supplementary material for: Revolutionizing market surveillance: customer relationship management with machine learning
Source: PeerJ Comput Sci. 2024 Dec 18;10:e2583. doi: 10.7717/peerj-cs.2583 (PMC11784820; doi:10.7717/peerj-cs.2583)
Supplement: Supplemental Information 3 [file peerj-cs-10-2583-s003.docx]

- **Operating System**: Ubuntu 20.04 LTS
- **Hardware**: Intel Core i7-9700K CPU @ 3.60GHz, 32 GB RAM, NVIDIA GeForce RTX 2070
- **Software**: Python 3.8, Jupyter Notebook, Anaconda Distribution
