# Supplementary material for: Revolutionizing market surveillance: customer relationship management with machine learning
Source: PeerJ Comput Sci. 2024 Dec 18;10:e2583. doi: 10.7717/peerj-cs.2583 (PMC11784820; doi:10.7717/peerj-cs.2583)
Supplement: Supplemental Information 4 [file peerj-cs-10-2583-s004.docx]

The SmartSurveil CRM model aims to predict customer churn with high accuracy, robustness, and flexibility. To achieve this, we carefully selected a combination of machine learning algorithms based on their strengths and suitability for handling complex customer behavior da

1. **Ensemble Learning Methods:**
   - **Random Forest:**
     - **Reason for Selection:**
       - Random Forest is an ensemble learning method that builds multiple decision trees and merges them together to get a more accurate and stable prediction.
       - It reduces the risk of overfitting, which is crucial when dealing with large and complex datasets like customer behavior data.
       - It can handle both categorical and numerical data and manage missing values effectively.
     - **Advantages:**
       - **Robustness:** By averaging multiple decision trees, Random Forest provides robust predictions.
       - **Feature Importance:** It can provide insights into which features are most important for predicting churn.
       - **Scalability:** It scales well with large datasets.
   - **Gradient Boosting:**
     - **Reason for Selection:**
       - Gradient Boosting builds models sequentially, each new model correcting errors made by the previous ones.
       - This method is effective in improving the accuracy of predictions by focusing on hard-to-predict cases.
     - **Advantages:**
       - **High Accuracy:** Gradient Boosting models often deliver high predictive performance.
       - **Flexibility:** It can optimize a variety of loss functions and provide several hyperparameters that can be fine-tuned to improve model performance.
       - **Handling Complex Patterns:** It can model complex relationships within the data by combining weak learners to form a strong predictor.
2. **Support Vector Machine (SVM):**
   - **Reason for Selection:**
     - SVM is known for its effectiveness in high-dimensional spaces, which is beneficial for customer behavior data that often involves numerous features.
     - It is particularly useful when the number of dimensions exceeds the number of samples, a common scenario in churn prediction.
     - SVM can also handle non-linear relationships by using kernel functions.
   - **Advantages:**
     - **High-Dimensional Data:** SVM performs well in high-dimensional spaces.
     - **Versatility:** Through the use of different kernel functions (linear, polynomial, RBF), SVM can model complex decision boundaries.
     - **Generalization:** It aims to find the hyperplane that best separates the classes (churn vs. non-churn) with maximum margin, improving generalization to new data.
3. **XGBoost (Extreme Gradient Boosting):**
   - **Reason for Selection:**
     - XGBoost is an optimized distributed gradient boosting library designed to be highly efficient, flexible, and portable.
     - It implements machine learning algorithms under the Gradient Boosting framework.
   - **Advantages:**
     - **Speed and Performance:** XGBoost is known for its speed and performance, making it suitable for large datasets.
     - **Regularization:** It includes L1 and L2 regularization to prevent overfitting, which is essential for churn prediction models.
     - **Handling Missing Data:** XGBoost has a built-in mechanism to handle missing data, making it robust for real-world datasets.

**Conclusion**

The choice of Random Forest, Gradient Boosting, SVM, and XGBoost for the SmartSurveil CRM model is motivated by the need to leverage the strengths of different algorithms to improve the accuracy and robustness of churn predictions. Ensemble learning methods like Random Forest and Gradient Boosting offer the advantage of combining multiple models to reduce overfitting and enhance prediction stability. SVM's capability to handle high-dimensional data and XGBoost's efficiency and performance further complement the ensemble, ensuring the model can effectively handle the complexities and nuances of customer behavior data.
