# Supplementary material for: Revolutionizing market surveillance: customer relationship management with machine learning
Source: PeerJ Comput Sci. 2024 Dec 18;10:e2583. doi: 10.7717/peerj-cs.2583 (PMC11784820; doi:10.7717/peerj-cs.2583)
Supplement: Supplemental Information 5 [file peerj-cs-10-2583-s005.docx]

#### Selection Method:

- **Random Forest**: Chosen for its robustness and ability to handle large datasets with high dimensionality. It reduces overfitting by averaging multiple decision trees.
- **Gradient Boosting**: Selected for its effectiveness in improving predictive performance through sequentially correcting the errors of weak classifiers.
- **Support Vector Machine (SVM)**: Included for its strength in classification tasks, especially in high-dimensional spaces, by finding the optimal hyperplane that maximizes the margin between classes.
