# Supplementary material for: Revolutionizing market surveillance: customer relationship management with machine learning
Source: PeerJ Comput Sci. 2024 Dec 18;10:e2583. doi: 10.7717/peerj-cs.2583 (PMC11784820; doi:10.7717/peerj-cs.2583)
Supplement: Supplemental Information 6 [file peerj-cs-10-2583-s006.docx]

Data preprocessing involved the following steps:

1. **Handling Missing Values**: Imputation of missing values using mean/mode for numerical/categorical features respectively.
2. **Encoding Categorical Variables**: Converting categorical variables such as gender into numerical format using one-hot encoding.
3. **Feature Scaling**: Standardizing numerical features to have zero mean and unit variance.
