# Supplementary material for: Revolutionizing market surveillance: customer relationship management with machine learning
Source: PeerJ Comput Sci. 2024 Dec 18;10:e2583. doi: 10.7717/peerj-cs.2583 (PMC11784820; doi:10.7717/peerj-cs.2583)
Supplement: Supplemental Information 7 [file peerj-cs-10-2583-s007.docx]

To ensure reproducibility, the entire modeling process, from data preprocessing to model training and evaluation, was implemented in Python using well-established libraries such as pandas, scikit-learn, and openpyxl. The code is documented and structured to allow easy replication of the results. Additionally, the dataset and code are shared in the supplementary materials.
